# Novel *gp60* subtypes and genetic diversity of *Cryptosporidium felis* in domestic and stray cats from Central Europe

**DOI:** 10.1186/s13071-025-07079-1

**Published:** 2025-11-21

**Authors:** Veronika Zikmundová, Eliška Höflerová, Nikola Holubová, Marta Kicia, Matúš Rajský, Pavel Beran, Bohumil Sak, Martin Kváč

**Affiliations:** 1 Institute of Parasitology, Biology Centre of the Academy of Sciences of the Czech Republic, V.V.I, Branišovská 31, 370 05 České Budějovice, Czech Republic; 2 Faculty of Agriculture and Technology, University of South Bohemia in České Budějovice, České Budějovice, Czech Republic; 3 Department of Biology and Medical Parasitology, Wroclaw Medical University, Wroclaw, Poland; 4 National Agricultural and Food Centre, Lužianky, Slovakia; 5 Faculty of Forestry, Technical University in Zvolen, Zvolen, Slovakia

**Keywords:** gp60, PCR, Cat, *Cryptosporidium*, Subtyping, Geographic distribution

## Abstract

**Background:**

*Cryptosporidium felis*, a host-specific protozoan with possible zoonotic potential, is a common parasite of cats (*Felis catus*). To date, there have been few studies on the molecular subtyping of *C*. *felis* worldwide, and no have been conducted in Central Europe. The aim of this study was to analyse the prevalence and genetic variability of *C*. *felis* in domestic and stray cats in Central Europe, particularly in the Czech Republic, Poland and Slovakia.

**Methods:**

Faecal samples were collected from domestic and stray cats. The presence of *Cryptosporidium* spp. was analysed by light microscopy with aniline-carbol-methyl violet staining and polymerase chain reaction (PCR)/sequencing of the small subunit rRNA gene (18S rDNA). All PCR-positive samples were further subtyped using PCR and sequencing of the 60 kDa glycoprotein gene (*gp60*). A chi-square test and odds ratio (OR) analysis were used to compare infection rates and assess the risk of *C*. *felis* infection between domestic and stray cats.

**Results:**

A total of 711 faecal samples were collected – 350 from domestic cats and 361 from stray cats. The overall infection rate of *Cryptosporidium* spp. was 4.5% (32/711), with stray cats being significantly more frequently infected (6.7%) than domestic cats (2.3%). Oocysts of *Cryptosporidium* spp. were not detected microscopically in any of the samples. There were no significant differences between the infection rates in the three countries. All isolates were identified as *C*. *felis*, and analysis of the *gp60* gene revealed five different subtypes, all belonging to the XIXa subtype family of *C*. *felis*. These subtypes formed five well-supported phylogenetic clusters, none of which had been previously reported worldwide. Only one subtype was found in domestic cats, whereas all five subtypes were found in stray cats. The subtypes identified in stray cats showed a clear geographical distribution in the study region.

**Conclusions:**

The results of this study extend our knowledge of the genetic variability of *C*. *felis* and indicate a possible geographical distribution of the detected subtypes. The significance of the observed genetic variability in terms of geographical distribution, host specificity and zoonotic potential remains unclear and requires further investigation.

**Graphical Abstract:**

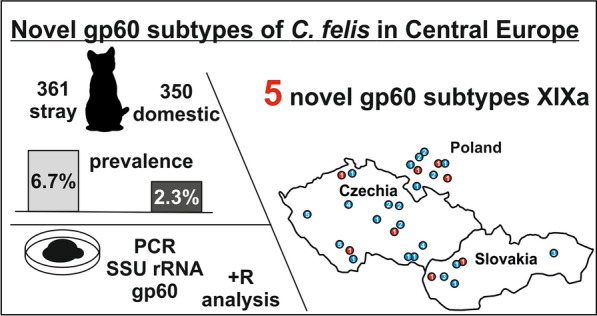

## Background

*Cryptosporidium* (Apicomplexa) is a genus of obligate parasitic protists that predominantly infect the gastrointestinal and respiratory tracts of vertebrates such as mammals, birds, reptiles, fish and amphibians [1–3]. A clinical manifestation of cryptosporidial infection, known as cryptosporidiosis, is characterised by watery diarrhoea, abdominal pain and nausea. The severity of infection depends on the *Cryptosporidium* species and the host species, including its age and immune status. In general, infection is more severe and sometimes fatal in juveniles, non-specific hosts and immunocompromised individuals [4–6].

The domestic cat (*Felis catus*) is one of the most widespread pet species and a favourite companion animal in many households worldwide. According to statistics, 350–370 million cats are kept as pets worldwide and the number of stray cats is estimated at 480 million [7]. Keeping cats has many benefits for humans – they improve mental well-being, reduce stress, promote socialisation and can have a positive effect on cardiovascular health [8]. However, the potential health risks must also be considered. Besides the usual injuries to the owner from scratching or biting and allergic reactions [9], cats can be carriers of various zoonotic parasite infections such as toxoplasmosis, giardiasis, or cryptosporidiosis [10–12].

Cats are primarily parasitised by the host-specific species *Cryptosporidium felis*, but other species and genotypes of *Cryptosporidium* have also been detected in cats, namely *Cryptosporidium muris*, *Cryptosporidium parvum*, *Cryptosporidium baileyi*, *Cryptosporidium ryanae*, *Cryptosporidium hominis*, *Cryptosporidium* sp. rat genotype III and IV [10, 13–16]. *Cryptosporidium felis* is classified as a species with zoonotic potential, with low to moderate prevalence in immunocompetent individuals or in populations with limited access to sanitation and clean water [17].

In 2020, Rojas-Lopez et al. [18] developed a molecular method for subtyping *C*. *felis* on the basis of the analysis of the gene encoding the 60 kDa glycoprotein (*gp60*). Phylogenetic analyses of this gene revealed the existence of five subtypes of *C*. *felis* (designated XIXa–XIXe), which differ both in their host specificity and geographical distribution [18–20]. Nevertheless, the amount of available data is still quite limited. Especially in the Europe, the genetic variation of *C*. *felis* is poorly documented, with few published data [10, 18–21].

The aim of the present study is to cover the knowledge gaps on the geographical distribution and genetic variability of *C*. *felis* in Central Europe by molecular subtyping based on the *gp60* gene.

## Methods

### Area and specimens studied

**Fig. 1:**
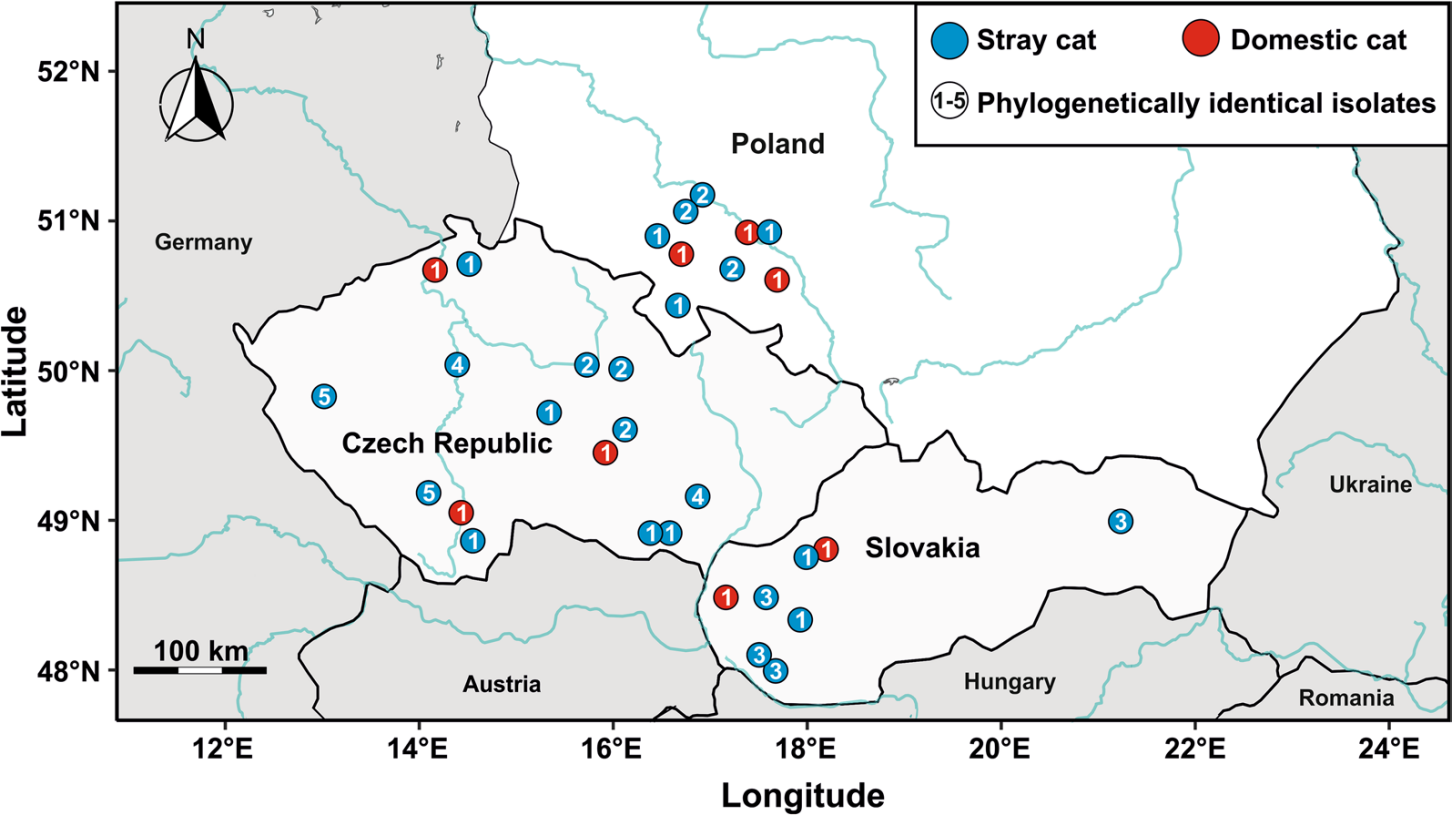
Map of the geographical distribution of phylogenetically identical *Cryptosporidium felis* subtypes (cluster) in domestic and stray cats in the Czech Republic, Slovakia and Poland, based on genotyping of the gene encoding the 60 kDa glycoprotein (*gp60*)

For this work, faecal samples were collected from domestic cats kept indoors, and from stray cats in animal shelters or from cats near farm buildings, especially cattle and pig stables. The samples were collected in the Czech Republic, Slovakia and the southern part of Poland (Fig. 1). To avoid re-sampling the same stray cat, samples were collected from the ground after the animal had defecated, within 1 day/location. No domestic cat was sampled twice. Each faecal sample was placed in a sterile plastic vial labelled with the sample ID and stored at 4–8 °C without fixative until laboratory processing. A smear was taken from each faecal sample, stained with aniline-carbol-methyl violet and examined using a light microscope to detect *Cryptosporidium* spp. oocysts [22].

### Molecular analyses

Total genomic DNA (gDNA) was extracted from 200 mg of faeces by bead disruption at 5.5 m/s for 60 s, using 0.5 mm glass beads in a FastPrepR24 instrument (MP Biomedicals, CA, USA), followed by DNA isolation using a commercial kit according to the manufacturer’s instructions (Exgene™ Stool DNA mini, Genially Biotechnology Co. Ltd., Seoul, Korea). The purified DNA was stored at −20°C before being used for PCR. A nested PCR approach was used to amplify a partial region of the small subunit rRNA gene (18S rDNA) [23] and the *gp60* gene [18]. The PCR reaction was carried out in a total volume of 20 µl. The reaction mixture contained 2 µl of gDNA as template, or 2 µl of the primary PCR product in case of a secondary reaction, 10 µl of 2 × AmpONE™ HS-Taq premix (GeneAll), 200 nM of each primer (forward and reverse) and molecular grade water to the total volume. *Cryptosporidium serpentis* (for 18S rDNA) and *C*. *felis* (for *gp60*) DNA and molecular grade water were used as positive and negative controls, respectively. The secondary PCR products were detected by agarose gel electrophoresis, stained with ethidium bromide and isolated using the GenElute Gel Extraction Kit (Sigma, St. Louis, MO, USA). The purified secondary products were sequenced bidirectionally at a commercial company (SeqMe s.r.o, Dobříš, Czech Republic) using Sanger sequencing. Each positive sample was independently sequenced twice.

### Phylogenetic analyses

The nucleotide sequences obtained for each gene in this study were processed and edited using ChromasPro v2.4.1 (Technelysium Pty Ltd., South Brisbane, Australia). Sequence alignments were performed both between samples and with reference sequences from GenBank, using the MAFFT online platform (version 7) with automatic alignment mode selection (http://mafft.cbrc.jp/alignment/software/). Phylogenetic analyses were performed using the MEGA X software [24, 25], which was also used to determine the best-fitting DNA/protein evolutionary models based on the Bayesian information criterion (BIC). The maximum likelihood (ML) method was applied to generate phylogenetic tree. The General Time Reversible model [26] was used for *gp60* tree. Branch support was evaluated using 1,000 bootstrap repetitions. The resulting phylograms were generated in MEGAX and then manually refined in CorelDraw X7. All newly generated sequences of *gp60* genes were deposited in GenBank under the following accession numbers [PV817847–PV817854].

### Statistical analysis

Statistical analysis was performed using chi-square tests with Yates correction in EpiInfo™ 7.2.7 (CDC, Atlanta, USA), to compare the frequency of *C*. *felis* occurrence between domestic and stray cats, and the distribution in each country (Czech Republic, Slovakia and Poland). A *P*-value < 0.05 was considered statistically significant.

## Results

### Occurrence and prevalence of *Cryptosporidium* spp. in cats

A total of 711 cat samples were collected – 350 from domestic cats and 361 from stray cats (Table 1). Of the 711 samples analysed, no oocysts of *Cryptosporidium* spp. were detected microscopically in any sample. However, the results of the molecular analyses for amplification of 18S rDNA showed the presence of specific *Cryptosporidium* spp. DNA in 32 samples (Table 1).

**Table 1:** Numbers of tested and positive domestic and stray cats (*Felis catus*) for the presence of *Cryptosporidium felis* in the Czech Republic, Slovakia and Poland

Country	Cat type	Examined	Positivity	
			*Microscopy*	*PCR*
Czech Republic	Domestic	141	0	3
	Stray	156	0	12
	Subtotal	*297*	*0*	*15*
Slovakia	Domestic	89	0	2
	Stray	102	0	6
	Subtotal	*191*	*0*	*8*
Poland	Domestic	120	0	3
	Stray	103	0	6
	Subtotal	*223*	*0*	*9*
Total		711	0	32

All PCR products were successfully sequenced, and the results confirmed the presence of *C*. *felis* in all samples. The overall prevalence of *C*. *felis* in the analysed regions was 4.5%. The highest prevalence was found in the Czech Republic (5.1%), followed by Slovakia (4.2%) and Poland (4.0%). Of the 350 domestic and 361 stray cats, 8 (2.3%) and 24 animals (6.7%), respectively, were positive for *C*. *felis*. Domestic cats were 3.04 times less likely to be parasitised by *C*. *felis* than stray cats (*P* = 0.0086, *χ*^*2*^ = 6.8863, *df.* = 1, OR = 3.04).

### Molecular subtyping of *Cryptosporidium felis*

**Fig. 2:**
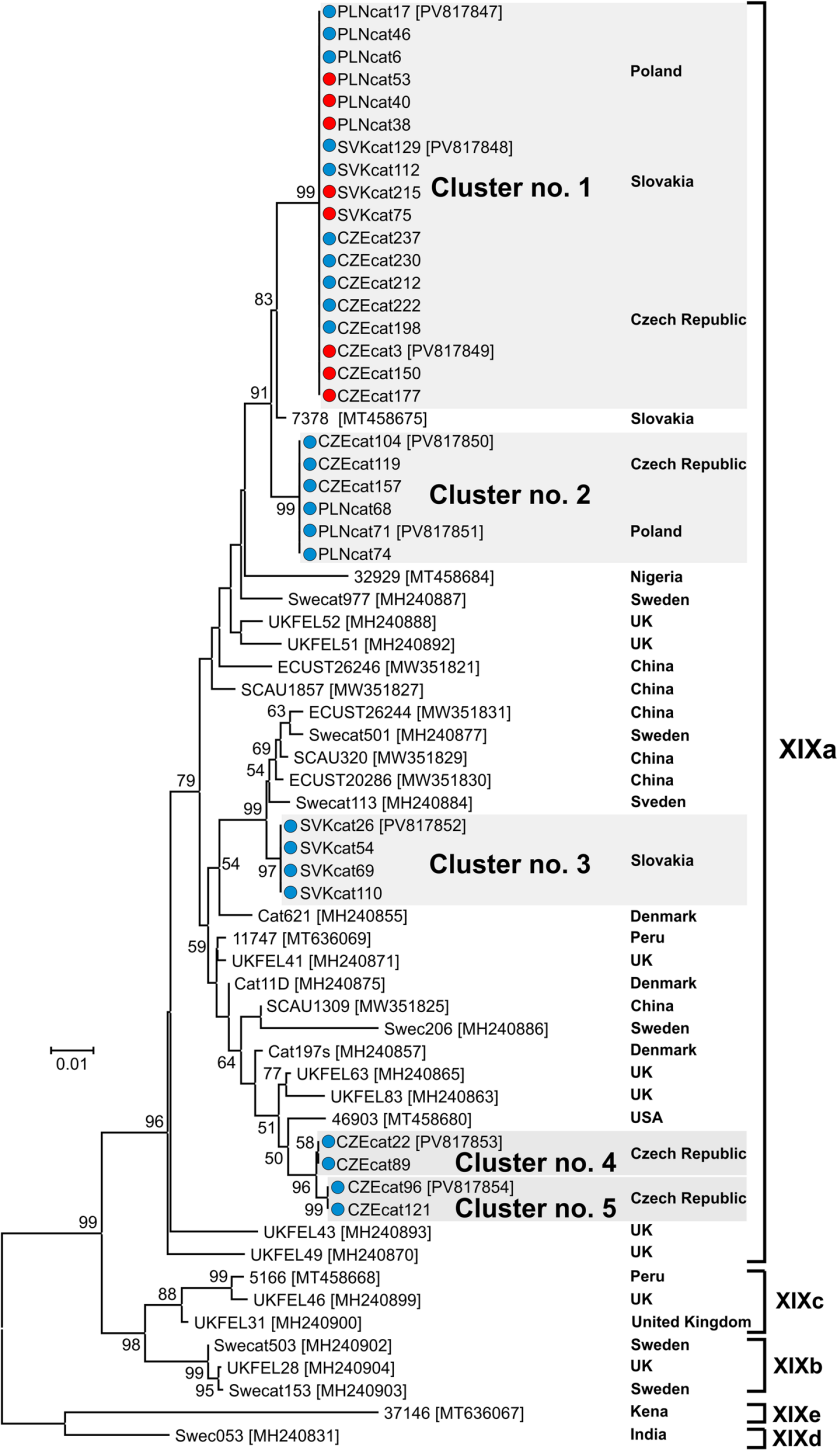
Phylogenetic relationships between *Cryptosporidium felis* isolates detected in this study and other *C*. *felis* subtypes, based on the partial sequence of the 60 kDa glycoprotein gene (*gp60*). Maximum likelihood analysis was performed using the General Time Reversible model. Numbers in the nodes represent bootstrap values for nodes that received more than 50% support in the bootstrap test after 1000 replicates. The scale bar is included in the tree. Isolates obtained in this study are highlighted in grey; red dots represent domestic and blue dots represent stray cats

All 32 PCR-positive samples were successfully genotyped for the *gp60* locus. Analysis of the *gp60* gene identified five subtypes of *C*. *felis*, which were assigned to subtype family XIXa (Fig. 2). When analysed phylogenetically, the subtypes formed five separate clusters with high bootstrap support. None of the subtypes detected in this study were identical to subtypes previously identified in cats or humans worldwide (Fig. 2). All isolates from domestic cats were identical to each other and belonged exclusively to cluster no. 1 (Fig. 2), which also included isolates from stray cats (Fig. 2). These isolates were phylogenetically related to isolate *C*. *felis* 7378 (GenBank MT458675), which originated from a cat in Slovakia (the type of cat is unknown), and to six other isolates obtained in this study from stray cats from Poland and the Czech Republic, which formed a separate cluster (no. 2). The analysis of the occurrence of the isolates in the study area indicates a geographical distribution pattern (Fig. 1). While isolates belonging to cluster no. 1 were detected in all three countries, the Czech Republic, Slovakia and Poland, isolates belonging to cluster no. 2 were found only in the Czech Republic and Poland. Cluster no. 3 was found exclusively in Slovakia, and clusters no. 4 and 5 only in the Czech Republic (Fig. 1 and 2).

Sequence analysis of the individual isolates revealed differences in sequence length, mainly due to the presence of repetitive sequences (Table 2). The sequence designated R1, which contains a 33-bp repetitive stretch of 5′ CCA CCT AGT GGC GGT GGC GTG TCC CCT GCT 3′ located approximately between nucleotides 450–530 in the sequence alignment, was present in only one copy in the isolates belonging to cluster no. 1 (Table 2; Fig. 2). A repetitive sequence designated R2, containing a 39-bp stretch of the tandem repeat 5′ AGC ACA ACT GCG GCT ACA GCG AGC ACT GCG AGT TCG ACA 3′ at a position between nucleotides 770–910, was detected in the sequences of eight isolates in repeats 1–3. Isolates with one tandem repeat belonged exclusively to cluster no. 3, those with two repeats to cluster no. 4 and those with three repeats to cluster no. 5 (Fig. 2; Table 2). The number of GTT triplet repeats at the end of the sequence, between nucleotide positions 1143–1154, was between two and four in the isolates analysed. Isolates belonging to clusters nos. 1 and 2 contained two GTT repeats, whereas isolates from clusters nos. 3–5 always had four repeats (Fig. 2; Table 2).

**Table 2:** The *Cryptosporidium felis* isolates detected in this study, their subtype identity and the copy numbers of major tandem repeats in the *gp60* gene

Country	Cat type	Isolate ID	Genotyping at *gp60*				Cluster
			Subtype	R1	R2	GGT	
Czech Republic	Domestic	CZEcat3	XIXa	1	–	2	1
	Domestic	CZEcat150	XIXa	1	–	2	1
	Domestic	CZEcat177	XIXa	1	–	2	1
	Stray	CZEcat22	XIXa	–	2	4	4
	Stray	CZEcat89	XIXa	–	2	4	4
	Stray	CZEcat96	XIXa	–	3	4	5
	Stray	CZEcat104	XIXa	–	–	2	2
	Stray	CZEcat119	XIXa	–	–	2	2
	Stray	CZEcat121	XIXa	–	3	4	5
	Stray	CZEcat157	XIXa	–	–	2	2
	Stray	CZEcat198	XIXa	1	–	2	1
	Stray	CZEcat212	XIXa	1	–	2	1
	Stray	CZEcat222	XIXa	1	–	2	1
	Stray	CZEcat230	XIXa	1	–	2	1
	Stray	CZEcat327	XIXa	1	–	2	1
Slovakia	Domestic	SVKcat75	XIXa	1	–	2	1
	Domestic	SVKcat215	XIXa	1	–	2	1
	Stray	SVKcat26	XIXa	–	1	4	3
	Stray	SVKcat54	XIXa	–	1	4	3
	Stray	SVKcat69	XIXa	–	1	4	3
	Stray	SVKcat110	XIXa	–	1	4	3
	Stray	SVKcat112	XIXa	1	–	2	1
	Stray	SVKcat129	XIXa	1	–	2	1
Poland	Domestic	PLNcat38	XIXa	1	–	2	1
	Domestic	PLNcat40	XIXa	1	–	2	1
	Domestic	PLNcat53	XIXa	1	–	2	1
	Stray	PLNcat6	XIXa	1	–	2	1
	Stray	PLNcat17	XIXa	1	–	2	1
	Stray	PLNcat46	XIXa	1	–	2	1
	Stray	PLNcat68	XIXa	–	–	2	2
	Stray	PLNcat71	XIXa	–	–	2	2
	Stray	PLNcat74	XIXa	–	–	2	2

Numbers of the clusters correspond to the clusters in Figs. 1 and 2

## Discussion

This study deals with the occurrence of *C*. *felis* in cats in the Czech Republic, Slovakia and Poland, focussing on the intraspecific variation within the *gp60* gene. *Cryptosporidium* spp. infections in cats are relatively common, and their prevalence in the population varies between different studies and countries, ranging from 0.6% to 40.8% [16]. In the present study, 4.5% of the 711 cats examined were classified as positive. This prevalence represents a relatively low incidence. Previous studies conducted in the Czech Republic, Slovakia and Poland found a similar prevalence of 4.4% [27]. Comparable prevalence rates were found in other European countries, with the exception of Germany, where an overall prevalence of 21.2% was observed [16].

The phylogenetic analyses showed that the cats included in the study were infected only with *C*. *felis*. These results are in agreement with those of other authors, who also either exclusively detected this species in their studies or dominated the occurrence of *C*. *felis* [10, 14, 19, 21, 28–33]. Sequence analysis of all 32 samples positive for *Cryptosporidium*-specific DNA showed no evidence of mixed infections, and all phylograms were clean. In a previous study conducted in the same area, no other *Cryptosporidium* species were detected in either stray or domestic cats [27]. The species *C*. *muris*, *C*. *parvum*, *C*. *ryanae*, *Cryptosporidium* sp. rat genotype III and *Cryptosporidium* sp. rat genotype IV, which have been detected together with *C*. *felis* in previous studies, mostly represent a group of species that are not host-specific to cats [2, 34]. The species and genotypes such as *C*. *muris*, *Cryptosporidium* sp. rat genotype III and *Cryptosporidium* sp. rat genotype IV are specific to mice and rats, which are a natural food source for cats [35, 36]. It is therefore not surprising that the specific DNA of these *Cryptosporidium* species is occasionally detected in cat faeces using sensitive molecular methods. Similarly, *C*. *muris* and *C. tyzzeri* have been detected in slurry and pig faeces; however, it was later experimentally confirmed that pigs are not susceptible to these *Cryptosporidium* species [37, 38]. The sporadic occurrence of *C*. *ryanae* in cats can be explained in a similar way. This *Cryptosporidium* species frequently infects cattle [39], it is likely that cats living on or near farms may become contaminated with cattle faeces.

Our findings, along with other studies, show that stray cats are more likely to be infected with *Cryptosporidium* spp. than domestic cats. In our dataset, the prevalence of *C*. *felis* infection was higher in stray cats (6.7%) than in domestic cats (2.3%). This is consistent with our previous study, in which 7.4% of stray cats were *C*. *felis*-positive, compared with only 0.8% in domestic cats [27]. Other studies investigating the influence of cat origin on the prevalence of *Cryptosporidium* spp. have reported similar trends. Yang et al. [15] showed that cats from refuge centres (13.4%) were more frequently infected than privately owned cats (7.1%). Similarly, Xu et al. [40] reported a higher prevalence in cats from animal shelters (7.5%) compared with cats from pet shops (3.8%).

Genotyping of *Cryptosporidium* spp. using the gene encoding *gp60* is frequently used to study intraspecific variation, host specificity and pathogenicity of subtypes within individual taxa. The *gp60* gene exhibits a high degree of polymorphism at both inter- and intraspecific levels, which enables the classification of individual isolates into specific families and subtypes within these families [18, 41–43].

Similar to other species, in particular *C*. *parvum*, *C*. *hominis* or *Cryptosporidium meleagridis*, a variation of the *gp60* gene has been described in *C*. *felis*, comprising five allelic families with dozens of subtypes [19]. In our study, we detected only isolates belonging to allele family XIXa, the most widespread allele family of *C*. *felis* worldwide. Previous studies suggest a possible geographical splitting of certain *C*. *felis* subtypes based on the occurrence of genetically identical clusters in geographical areas [19].

In line with these findings, our study not only detected new subtypes not previously reported, but also identified three clusters that appear to be geographically specific. However, it should be emphasised that these findings are based on limited data, in contrast to the well-studied species *C*. *parvum* or *C*. *hominis*. Further studies with larger samples size and in other countries are needed to draw more robust conclusions.

The significance of the diversity of *C*. *felis* subtypes in relation to pathogenicity – as described for *C*. *parvum* or *C*. *hominis*, for example – remains unclear [44–47]. To date, no studies have demonstrated an influence of a specific subtype on the clinical course of *C*. *felis* infection in cats or humans. In our study, we observed no clinical signs of cryptosporidiosis in any of the positive cats. However, it should be noted that all cats involved in the study were adults, which is consistent with previous findings that adult and healthy cats shed only small amounts of oocysts into the environment after infection [15, 48–51]. This is also consistent with our microscopically negative results, as the low level of oocyst excretion is below the detection limit of conventional staining methods, whereas PCR analyses detected specific *C*. *felis* DNA in certain samples.

Previous studies have shown that the subtype families XIXb, XIXd and XIXe have been detected exclusively in humans, whereas some subtypes from the families XIXa and XIXc have zoonotic potential [18–21]. Similar differences in the host specificity among subtype families and their subtypes have also been described for *C*. *parvum* or *Cryptosporidium equi*. For example, the subtypes of subtype family IIc of *C*. *parvum* are mainly found in humans, with no known natural hosts other than hedgehogs [52, 53]. A similar situation was observed with *C*. *equi*. While the subtypes of subtype family VIa occur exclusively in horses, donkeys and other equids, infections with subtypes from families VIb and VIc are almost exclusively associated with humans [54]. Since new subtypes of subtype family XIXa were detected in the countries investigated, and no cases of *C*. *felis* infections in humans have been reported from these areas, the zoonotic potential of these subtypes of family XIXa cannot yet be assessed.

## Conclusions

This study expands the current knowledge on the intraspecific diversity and distribution of *C*. *felis* in Central Europe. The detection of new subtypes of subtype family XIXa and evidence of a possible geographical clustering emphasise the need for further studies to clarify the zoonotic potential and biological significance. The higher prevalence in stray cats compared with domestic cats emphasises the role of lifestyle and environmental exposure in transmission dynamics. Finally, the discrepancy between microscopically negative and PCR-positive results emphasises the importance of molecular methods for reliable detection.

## Data Availability

All DNA material and datasets on which the conclusions of the manuscript rely are stored at the Institute of Parasitology, Biology Centre, Czech Academy of Sciences, České Budějovice, Czech Republic. Representative nucleotide sequences generated in this study were submitted to the GenBank database under the accession numbers [PV817847–PV817854].

## Abbreviations

BIC: Bayesian information criterion
DNA: Deoxyribonucleic acid
gDNA: Genomic DNA
*gp60*: 60 KDa glycoprotein gene
ML: Maximum likelihood
PCR: Polymerase chain reaction
rRNA: Ribosomal Ribonucleic Acid
OR: Odds ratio
18S rRNA: Small subunit rRNA
18S rDNA: Small subunit rDNA
